# Interpretable machine learning for river salinity dynamics in arid basins

**DOI:** 10.1038/s41598-026-49042-9

**Published:** 2026-04-20

**Authors:** Hossein Amini, Reza Shakeri, Narjes Ghaderi, Farshid Fakheri, Khosro Morovati, Banafsheh Zahraie, Reza Ahmadian

**Affiliations:** 1 Hydro-Environmental Research Centre, School of Engineering, Cardiff University, Cardiff, Wales, UK; 2 School of Civil Engineering, University of Tehran (UT), Tehran, Iran; 3 Department of Civil and Environmental Engineering, Amirkabir University of Technology, Tehran, Iran; 4 Department of Hydraulic Engineering, Tsinghua University, Beijing, 100084 China; 5 School of Civil Engineering, College of Engineering, University of Tehran, Tehran, Iran

**Keywords:** Salinity dynamics, Hydrological regimes, Water quality management, Arid rivers, Interpretable machine learning, Climate sciences, Environmental sciences, Hydrology

## Abstract

Managing salinity in arid rivers is impeded by sparse monitoring, relying on low-frequency grab samples that miss hydrological event dynamics. Here, interpretable machine learning is applied to a 50-year monthly archive (1968–2018; Discharge, major ions, pH) from three stations on Iran’s Karkheh River. Gradient Boosting Regression achieves high predictive skill for Total Dissolved Solids (TDS)/Electrical Conductivity (EC) (test-set R^2^ = 0.94/0.97; RMSE = 55 mg L^−1^/56 µS cm^−1^), validated via time-aware cross-validation. SHAP-based feature attribution reveals that Na^+^ and SO_4_^–2^ are the strongest contributors to TDS, while Na^+^ and Cl^−^ dominate EC, consistent with conservative salinity sources under baseflow conditions. A reduced-input decision tree (four predictors) retains R^2^ = 0.81–0.87, enabling minimal-sensor monitoring. Flow-regime partitioning and STL (Seasonal-Trend decomposition using Locally estimated scatterplot smoothing)-detrended event composites reveal low-flow salinization and ion-specific post-flood recovery (Cl^−^: 1–2 months; Na^+^: 2–3 months), guiding targeted sampling. This framework extracts predictive power, process associations, and operational guidance from legacy grab-sample archives, scalable to data-limited basins worldwide.

**Supplementary Information:**

The online version contains supplementary material available at 10.1038/s41598-026-49042-9.

## Introduction

Salinity dynamics and water scarcity in arid and semi-arid rivers are shaped by interacting climatic, hydrological, and anthropogenic pressures, yet monitoring networks often rely on low-frequency grab samples and sparse sensor deployments^1–4^. Long-term archives of monthly Discharge and ion chemistry are common in such systems but are rarely exploited to quantify how flow regimes and hydrologic events jointly control Total Dissolved Solids (TDS) and Electrical Conductivity (EC) over decadal time scales. Ensuring access to safe and reliable water supplies is fundamental to public health and environmental protection, yet the limited, seasonal nature of surface waters in these regions presents significant management challenges^5–9^.

Understanding the dominant hydrological processes governing salinity is essential for improving monitoring strategies. Indicators such as TDS and EC reflect dissolved salt concentrations influenced by flow regimes, evaporation, and groundwater inflow^10,11^. In arid basins, these controls operate through Ground Water-Surface Water (GW-SW) interfaces: baseflow and evaporative concentration elevate conservative ions (e.g., Na^+^, Cl^−^) during dry periods, whereas high-flow runoff enhances dilution. These pathway shifts alter solute transport and aquatic habitat quality; thus, flow regimes serve as practical proxies for attributing salinity dynamics. Elevated salinity levels compromise health and agriculture, signaling environmental degradation. While the WHO recommends specific acceptable limits for TDS and EC, process-based models often fail in these regions due to extreme data scarcity and computational demands^12–17^.

In contrast, data-driven Machine Learning (ML) offers a flexible approach to uncover nonlinear relationships. However, the “black-box” nature of many ML methods hampers the mechanistic understanding of underlying hydrological controls. Furthermore, standard ML approaches often overlook episodic variations, such as sudden salinity spikes, which are crucial for effective resource management^18–31^. To address this gap, we incorporate an anomaly detection framework to identify rare or abrupt changes in water quality. Integrating anomaly detection with interpretable ML provides a comprehensive understanding of both normative and extreme behavior. Leveraging a 50-year record from the Karkheh River, Iran, this study: (i) predicts TDS and EC using time-aware validation, (ii) identifies a minimal set of variables for cost-efficient monitoring, and (iii) analyzes flow regimes to quantify how floods and low-flow conditions drive salinity anomalies.

## Materials and methods

### Study area

**Fig. 1:**
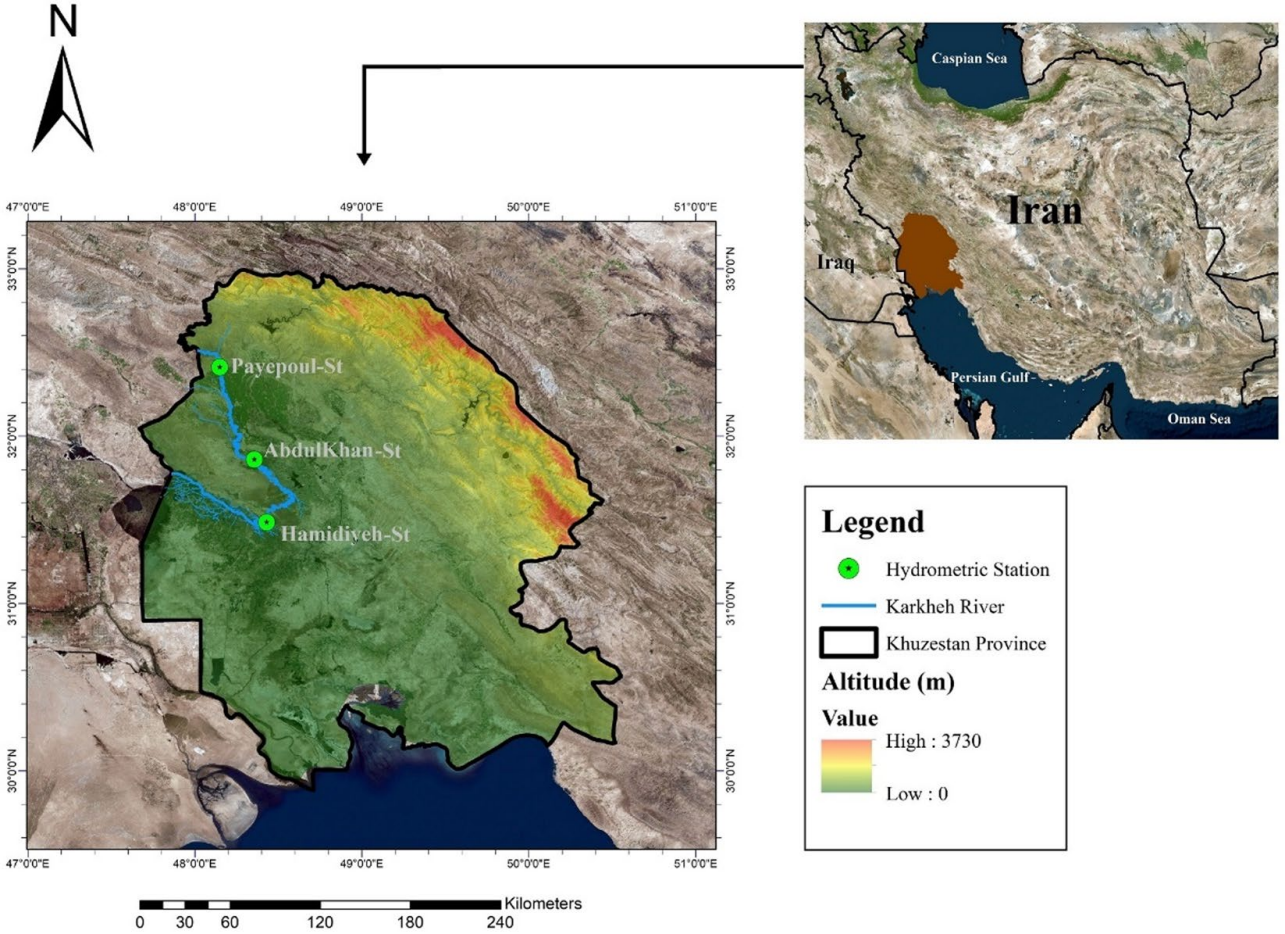
Location of the study area in Khuzestan Province, Iran, and the course of the Karkheh River. The main panel shows the province boundary, elevation (m), the Karkheh River network, and the locations of the hydrometric stations (Payepoul, Abdul Khan, and Hamidiyah). The inset map indicates the position of Khuzestan Province within Iran.

The Karkheh River in southwestern Iran was selected as the study area because it provides an exceptionally long and continuous 50-year record of hydrological and hydrochemical observations with minimal data gaps, enabling robust data-driven analysis. In addition, the basin is a strategically important hub for irrigation, drinking-water supply, and regional food and water security, which makes understanding its salinity dynamics particularly critical for sustainable water resources management. Its selection is further justified by significant changes in water quality indicators driven by climate change, urbanization, and anthropogenic activities, making it suitable for data-driven modeling^32^. The Karkheh, Iran’s third longest river at ~ 950 km, flows from the Zagros Mountains to the Persian Gulf via the Horul Azim marsh, with a long-term mean annual Discharge of approximately 185 m^3^/s (1961–2001, in Payepoul station)^33^ and a catchment area of ~ 46,000 km^2^. In a semi-arid region with 380 mm annual rainfall, 3000 mm evaporation, 18 °C average temperature, and 40% humidity, the river’s elevation ranges from 3000 to 500 m^34^. It supports irrigation, drinking water, and industry, significantly impacting regional socio-economic aspects^35^. The study area is shown in Fig. 1.

### Data preparation

All monthly data related to quality variables including TDS, EC, pH, cations: Sodium (Na^+^), Magnesium (Mg^+2^), Calcium (Ca^+2^) and anions: Chloride (Cl^−^), Sulfate (SO_4_^−2^), Bicarbonates (HCO_3_^−^) and Discharge were collected from three hydrometric stations located in Karkheh River (in Khuzestan province) between 1968 and 2018 (50 years) from the Ministry of Energy of Iran. This information was determined by a combination of field sensor methods and laboratory investigations. The geographical location of the stations is as follows: Hamidiyeh: 48° 25′ E and 31° 29′ N, Abdulkhan: 48° 21′ E and 31° 51′ N, Payepoul: 48° 08′ E and 32° 24′ N.

Due to possible monitoring gaps, some monthly records were missing for a few indicators in certain years. However, missing data accounted for less than 5% of the total dataset and occurred only in isolated months with short gaps and no consistent pattern across stations or variables. These gaps were filled using linear interpolation based on the closest available observations, applied before splitting the data into training and testing sets to avoid data leakage. Given the small proportion of missing values and their negligible effect on the overall results, an imputation sensitivity analysis was deemed unnecessary. Also, statistical methods commonly used in ML were then employed to examine the normality of the data.

Table 1 shows the monthly values of selected water quality variables of the Karkheh River and their comparison with the WHO guideline values for drinking water. It should be noted that the WHO values are guidelines and acceptability limits, and they are not legally enforceable standards.

**Table 1:** Monthly water quality variables of the Karkheh River compared with the WHO guideline values for drinking water.

Variable	Average values of three stations				Max acceptable value (WHO)	Max allowed Value (WHO)
	Min	Max	Mean	Standard deviation		
TDS (mg/l)	344.3	1601	857.2	231.5	600	1000
EC (µs/cm)	613	2446	1316.4	360.1	500	1500
Na (mg/l)	27.6	326.6	128.8	57.5	200	200
Mg (mg/l)	12.2	60.8	32.8	9	50	150
Ca (mg/l)	50.1	250.5	100.2	26	75	200
pH	5.3	8.5	7.9	0.26	6.5–8.5	
Cl (mg/l)	33.6	521.1	195	88.6	200	600
SO_4_ (mg/l)	81.6	561.8	245	81.6	200	400
HCO_3_ (mg/l)	67.1	244.1	164.8	26.2	150	350

### Methodology

**Fig. 2:**
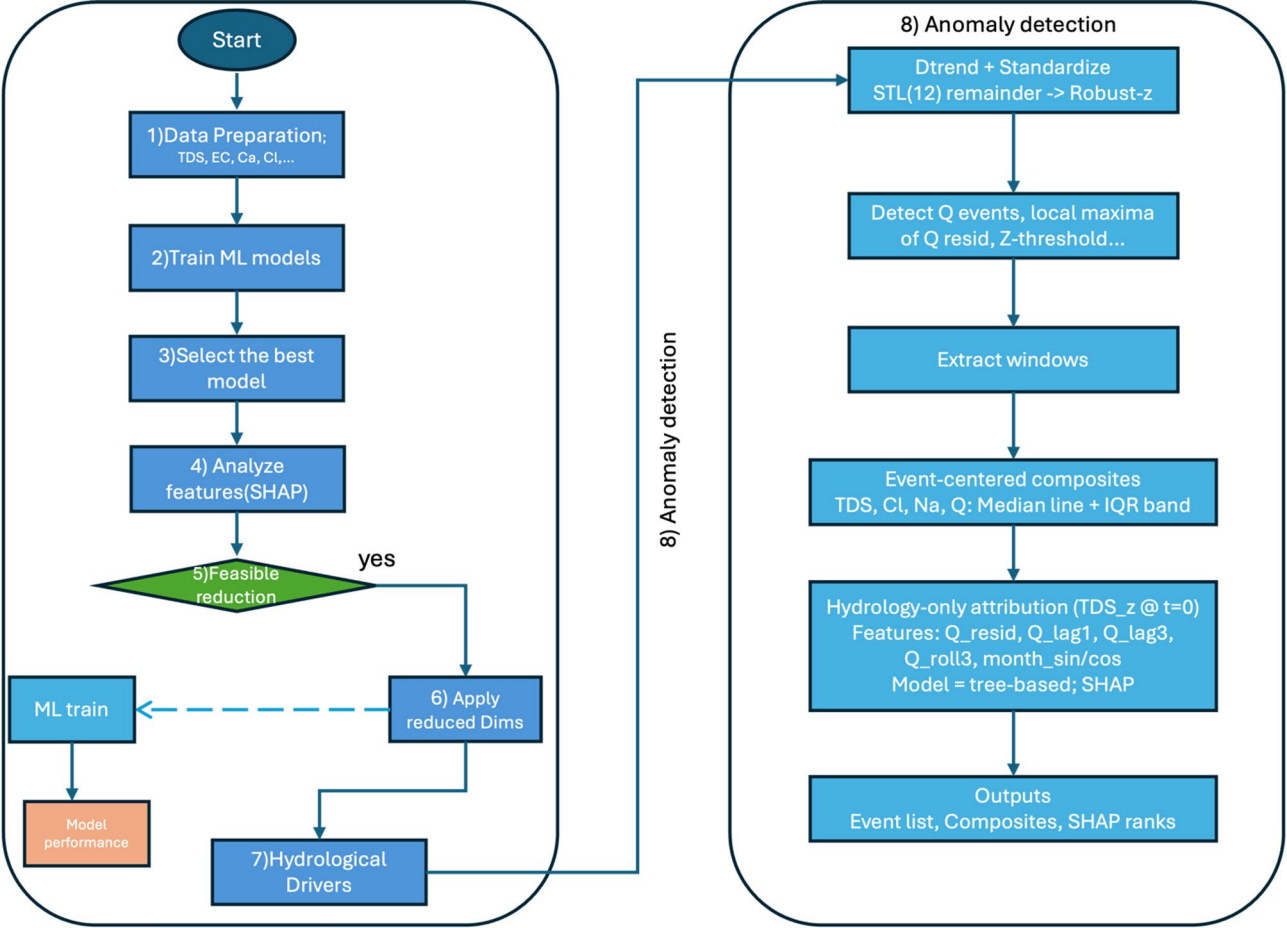
Workflow of the study. The left panel summarizes the predictive modelling pipeline (data preparation, model training and selection, interpretation using SHAP, and feature reduction using recursive feature elimination) used to identify key hydrological drivers of salinity indicators. The right panel shows the event-centred anomaly-detection framework based on detrended residual discharge, which detects high-flow events and quantifies associated dilution and recovery patterns in major ions and TDS through event-centred composites.

TDS and EC indicators were considered as dependent variables in ML, and the relation of cations, anions, pH, and discharge as independent variables with the two above indicators was investigated. The algorithms that were used in this work are Linear Regression as a linear algorithm and six non-linear algorithms, including Multi-Layer Perceptron (MLP), K_Nearest_Neighbor (KNN), Random_Forrest (RF), Decision_Tree (DT), and Gradient_Boosting methods (eXtreme_Gradient_Boosting (XGBoost) and Gradient_Boosting_Regression (GBR)) (all written in Python). These methods cover the most widely used non-linear algorithms, including tree-based algorithms, gradient boosting algorithms, and the most commonly used (MLP) algorithms. Finally, the relationship between the algorithms and the above indicators was analyzed and discussed (including prediction, dimensionality reduction, and variable importance), as well as identifying the best algorithm. The flowchart for the steps followed through this research is depicted in Fig. 2. In this flowchart, the first section is “Predictive modeling” and the second part is “Anomaly Detection”. In the first section, we first apply ML models and dimensionality reduction, and in the second part, we implement “Anomaly detection” to find the regime influence on TDS prediction.

#### Overview of machine learning models

A range of widely used ML models was employed to predict TDS and EC from hydrological and hydrochemical variables. These models were selected to cover a diverse set of nonlinear learners with varying levels of complexity and interpretability, suitable for capturing intricate relationships between Discharge, solutes, and salinity indices. In other words, the selection of ML architectures was driven by the need to balance predictive performance with structural diversity, allowing us to capture both linear and complex non-linear salinity drivers. We deployed different distinct algorithms, categorized by their underlying mathematical logic, to ensure that our findings are robust across different inductive biases^36^. Each model was trained and evaluated using cross-validation, with hyperparameters optimized via grid search to enhance predictive accuracy. Hyperparameter tuning was performed via time-blocked cross-validation on the training set only (using fivefold blocked splits preserving temporal order), with final models evaluated on the held-out chronological test set. While accurate prediction was important, the primary focus of this study was the interpretation of model outputs in the context of hydrological processes, particularly via variable importance and salinity dynamics under different flow regimes. Detailed descriptions of model architectures, parameter settings, and training procedures are provided in the Supplementary Document (S. 1–S. 5). The process of splitting into train and test is done to avoid overfitting and potential bias. Usually, 70 and 30 percent of the data are assigned to the train and test stages, respectively, which has been done in this study as well. By enabling future observations to inform model training, random splitting may induce temporal leakage given the dataset’s 50-year monthly structure. Leakage-safe time-series validation was used to re-evaluate model performance in order to guarantee strong temporal causality.

In particular, we put in place a blocked holdout strategy wherein 30% of the observations in chronological order were set aside for testing, and the first 70% were used for training. Furthermore, forward-chaining (rolling-origin) cross-validation was used, in which models were assessed on later unobserved temporal blocks after being successively trained on growing historical windows. To avoid information leakage, all preprocessing operations (including scaling) were carried out inside each training fold and applied to the matching validation fold. Therefore, purely out-of-sample, temporally ordered evaluation is reflected in reported performance indicators.

Note that while TDS and EC are compositionally related to major ions, the prediction task here integrates hydrological covariates (Discharge) to capture dilution and source-mixing effects, enabling dimensionality reduction to minimal proxy sets suitable for sparse monitoring networks. In data-limited scenarios prevalent in arid basins like the Karkheh, Discharge is often continuously available while ion/TDS/EC measurements rely on costly grab samples. The interpretable models thus support gap-filling, hindcasting during unmonitored events, and prioritization of key parameters for future sensor deployment. Also, it is important to point out that the interpretability architecture in this is used for the sake of avoiding treating the ML model as a “Black Box” and this is not meant causality, but rather it is the “Contributions of the features to every single prediction” that is made by the model.

In order to consider the dimensionality reduction, there are plenty of algorithms that have pros and cons, and, in this study, “Recursive Feature Elimination (RFE)” has been employed. Finally, reproducibility is an important aspect of AI-based modeling in environmental issues. In this study, the selection of the hyperparameters (including Boolean choices, or numeric choices) of the methods has been declared as the architecture of the ML models (Tables 1, 2, 3, 4, 5 in the supplementary document).

#### Hydrological regime analysis

Flow regimes were classified into low, normal, and high flows based on the 25th and 75th percentiles of discharge, reflecting the river’s seasonal variability. Low flows correspond to baseflow-dominated conditions with potential groundwater inflows and evaporation, while high flows indicate surface runoff and dilution capacity. This classification captures the study area’s hydrological dynamics, shaped by arid climate and anthropogenic pressures such as irrigation and urbanization^10^. The hydrological regime analysis links flow conditions to salinity dynamics. During high flows, increased Discharge dilutes TDS and EC, reducing ion concentrations (e.g., Environmental variables). Conversely, low flows amplify salinization due to groundwater contributions, evaporation, and anthropogenic inputs like agricultural runoff^33^. These findings are supported by ML results and anomaly detection results.

Monthly time series were de-seasonalized and de-trended using Seasonal-Trend decomposition via Locally estimated scatterplot smoothing (STL; period = 12), yielding robust z-score residuals (median = 0, MAD-scaled). Hydrologic events were identified as local maxima in Discharge residuals exceeding a z-threshold of ± 2.0 (*N* = 65 events across 1968–2018). For each event, 6-month (± 3 month) windows were extracted to construct median composites with bootstrap 95% CIs (*n* = 1000). Robustness was assessed via sensitivity analyses: z-thresholds 1.5–2.5 (event counts: 92–42); window lengths 4–8 months (recovery timescale stability ± 0.5 months); STL period 10–14. Composites remained qualitatively consistent (peak dilution amplitude − 0.5 to − 1.2 z; recovery 1–3 months), with *N* = 65 (± 2.0) yielding optimal signal-to-noise. Multivariate profiles (Discharge, TDS, Cl, Na residuals) were clustered (k = 2–5; Silhouette/Calinski-Harabasz optimal k = 3), distinguishing dilution vs mixed regimes. TDS anomaly attribution used a hydrology-only ARIMA model (Discharge residual + 1/3-month lags + harmonics; CV-R^2^ = 0.62) with SHAP to avoid ion leakage. Imputation impact was tested by excluding/re-running on complete-case periods (> 95% observed data): event detection yielded *N* = 58 (vs 65 full), with near-identical composites (Δmedian < 0.1 z).

Finally, the performance of used models was compared using standard statistical metrics, including: (1) Root Mean Square Error or RMSE, (2) Mean Absolute Error or MAE, (3) Coefficient of Determination or R^2^, and (4) Pearson Correlation Coefficient or R. The equations of the mentioned metrics are given below.

1 $$RMSE = \sqrt { \frac{1}{n} \mathop \sum \limits_{i = 1}^{n} (y_{o} - y_{p} )_{i}^{2} }$$

2 $$MAE = \frac{1}{n} \mathop \sum \limits_{i = 1}^{n} \left| {(y_{o} - y_{p} )} \right|_{i}$$

3 $$R^{ 2} = 1 - \frac{RSS}{{TSS}} = \frac{{\sum \left( {\hat{y}_{p} - \overline{y}_{o} } \right) ^{2} }}{{\sum (y_{o} - \overline{y}_{o} ) ^{2} }}$$

4 $$R = \rho_{XY} = \frac{{Cov \left( {X,Y} \right)}}{{ \sigma_{x} \sigma_{y} }}$$

where *n* represents the total number of observed data points. The term $y_{o}$ denotes the observed data, while $y_{p}$ refers to the data predicted (or simulated) by the ML models. The Residual Sum of Squares (RSS) is the sum of the squared differences between the observed and predicted values. The Total Sum of Squares (TSS) measures the total variance in the observed data. The predicted value of the observed data is indicated by $\hat{y}_{p}$ and the mean of the observed data is represented by $\overline{y}_{o}$. *R* or $\rho_{XY}$ is the Pearson’s coefficient, $Cov \left( {X,Y} \right)$ is the covariance between two generic variables *X* and *Y*, and $\sigma$ is the standard deviation of two variables. The closer the RMSE and MAE values are to zero and the closer the R^2^ value is to one, the more accurate the model is. The R coefficient ranges from − 1 to + 1, with values close to + 1 indicating a strong positive correlation, values near − 1 indicating a strong negative relationship, and values around zero suggesting no correlation^37^.

## Results and discussion

### Correlation analysis

**Fig. 3:**
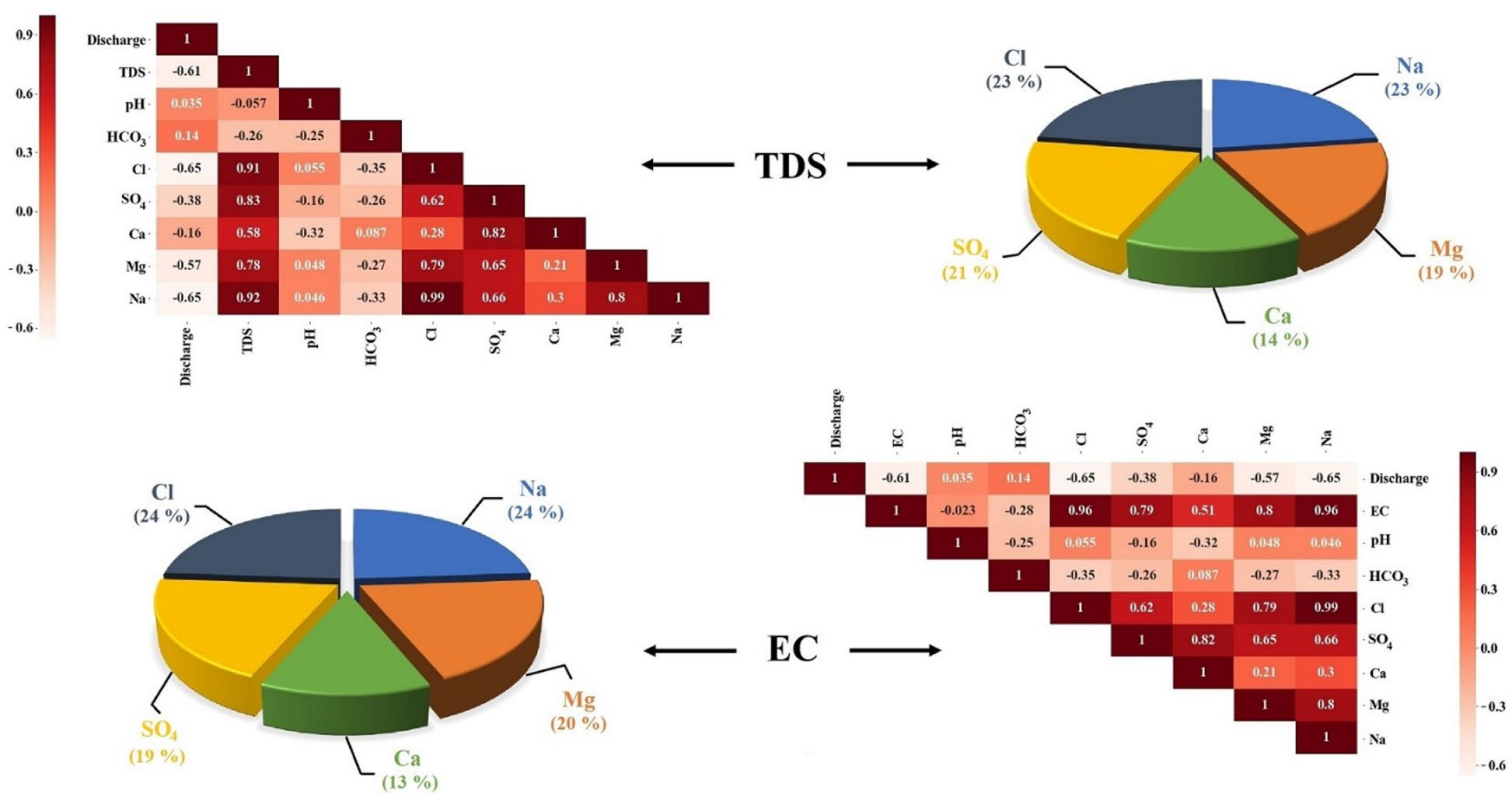
Pearson correlation matrix among Discharge, major ions (Na, Mg, Ca, Cl, SO₄, HCO₃), pH, and salinity indicators (TDS and EC) for the Karkheh River (1968–2018). The panels show correlation heatmaps highlighting pairwise linear relationships, with color intensity representing correlation strength (R) and pie charts display the relative proportional contributions of positively correlated ions to TDS (top) and EC (bottom), illustrating the dominant compositional associations of Na and Cl with salinity indicators.

The correlation of all investigated variables with respect to the two TDS and EC indicators was assessed according to the Pearson correlation coefficient (R) method, and these results and a pie chart of variables positively correlated with TDS and EC are presented in Fig. 3. It can be seen that Na has the most correlation (+ 0.92) and Ca has the least correlation (+ 0.58) for the TDS indicator. The two components of Cl and Na, with a correlation coefficient of + 0.99, and the two components of pH and Discharge, with a correlation coefficient of 0.035, have the greatest and least correlation with each other, respectively. Na, Cl, and Ca components have the most and least correlation on EC, with correlation coefficients of 0.96 and 0.51, respectively. Based on the pie chart, Na and Cl showed the strongest positive correlation with TDS and EC, followed by SO_4_ with TDS and Mg with EC.

### Machine learning modeling performance

**Fig. 4:**
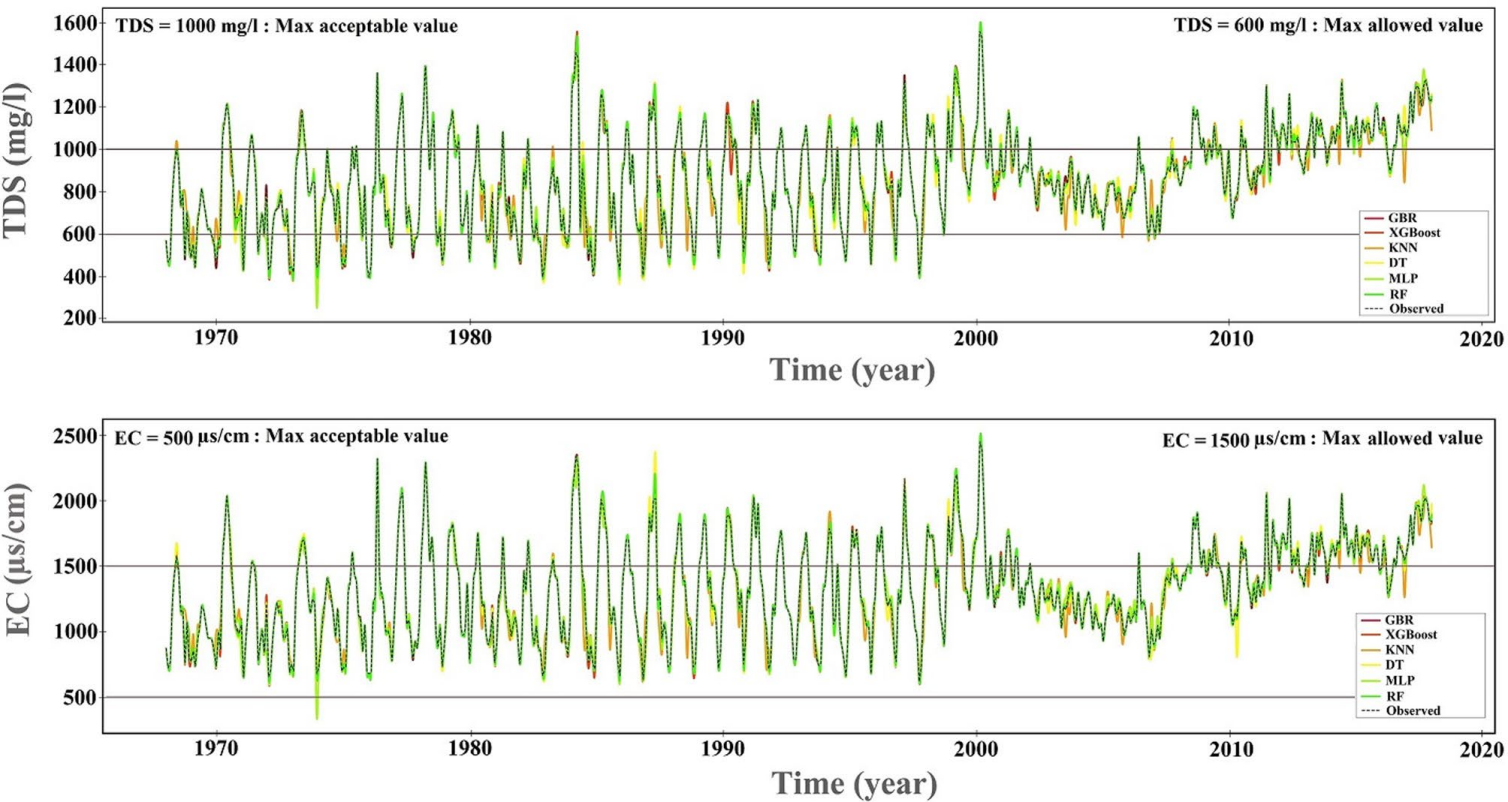
The Comparison of observed and ML-predicted monthly TDS and EC (1968–2018). WHO guideline limits are shown as horizontal reference lines. Multiple nonlinear models reproduce the long-term variability of both salinity indicators.The big jump in the TDS/EC average from early 2000 is related to the opening of the deployability of Karkher dam.

Due to the nature of non-linear models, they usually perform better than linear models. Figure 4 highlights that the concordance ML models and the measured data are good, and ML models could predict measured data accurately. The values of RMSE, MAE, and R^2^ for TDS and EC are given in Table 2. All the used models, except the KNN model, perform well in relation to TDS and EC, and their R^2^ are almost all above 0.9. The GBR model with RMSE = 54.85, MAE = 41.02, and R^2^ = 0.94 has the highest accuracy, and the KNN with RMSE = 113.18, MAE = 88.20, and R^2^ = 0.76 has the weakest performance (lowest accuracy) in relation to TDS. Again, about EC, the models of GBR (RMSE = 55.73, MAE = 41.9, R^2^ = 0.97) and KNN (RMSE = 141.36, MAE = 109.31, R^2^ = 0.83) have the best and worst performance in terms of the accuracy model. These highlight that the different algorithms used here, apart from the KNN model, could predict EC and TDS.

**Table 2:** The Performance of models for EC and TDS; values of RMSE, MAE, and R^2^ for ML models used.

ML models	TDS			EC		
	RMSE	MAE	R^2^	RMSE	MAE	R^2^
MLP	62.01	46.76	0.93	77.76	62.47	0.94
KNN	113.18	88.20	0.76	141.36	109.31	0.83
RF	55.82	41.42	0.94	59.33	43.60	0.97
DT	73.52	58.04	0.88	85.17	61.01	0.95
XGBoost	62.42	47.05	0.92	61.41	47.21	0.96
GBR	54.85	41.02	0.94	55.73	41.90	0.97

We took a step forward towards variable importance and interpretability, and studied the feasibility of dimensionality reduction. These new applications of ML models are for avoiding prediction with wrong training, to not use ML as a black box. Through interpretability, it is possible to realize the importance of the variables in the prediction that is made in the ML model.

The robustness of performance across folds under rolling-origin cross-validation confirmed that temporal leakage did not drive model competence. For both EC and TDS, the distribution of R^2^ values among forward-chaining folds (Supplementary document, Fig. S.4) shows consistent predictive efficacy. The models continuously obtained much higher R^2^ under blocked holdout evaluation (particularly the GBR model) when compared to naïve baselines (mean and persistence models), demonstrating true predictive ability beyond temporal autocorrelation effects (Supplementary document, Fig. S.5). These results validate that actual out-of-sample forecasting ability, not information leakage, is reflected in the reported model performance. Although the linear models achieved competitive predictive performance, suggesting that a substantial component of EC/TDS variability is governed by structured, near-linear compositional relationships among major ions. However, tree-based models were retained to allow flexible interaction modeling and feature-attribution analysis under strict time-aware validation.

The long-term TDS and EC time series for the Karkheh River (1968–2018) show pronounced interannual variability superimposed on a clear upward trend over roughly the past decade (from about 2008 onward), with recent values frequently approaching or exceeding guideline thresholds (Fig. 4). These increases are plausibly linked to intensified anthropogenic pressures in Khuzestan including expanded irrigation, industrial development, and urban growth which enhance return flows and runoff carrying dissolved salts and other pollutants to the river.

In parallel, climate-driven changes such as higher air temperatures and altered precipitation regimes likely exacerbate salinity by increasing evaporation, concentrating dissolved solids, and reducing the river’s capacity to dilute contaminant inputs. Together, these human and climatic drivers provide a coherent explanation for the observed rise in TDS and EC and underscore the need for sustainable water-resources management and targeted mitigation measures in the basin.

### Dimensionality reduction

Table 3 shows how data can be explained in different models by reducing the dimensions. These results are summarized in Table 3 and demonstrate that substantial dimensionality reduction can be achieved with minimal loss in the explainability of the data in different ML models, confirming the effectiveness of feature selection and the robustness of the modeling approach. Among the three ML models, the DT model performed best, reducing eight variables to four for both TDS and EC.

**Table 3:** The number of reduced variables in the DT, XGBoost, and GBR models.

ML models	TDS			EC		
	Current variables	Reduced variables	Score	Current variables	Reduced variables	Score
DT	8	4	0.88	8	4	0.95
XGBoost	8	6	0.92	8	7	0.96
GBR	8	6	0.94	8	6	0.97

The results related to the dimensionality reduction for the DT as the best model show that the reduced feature set yielded an RMSE of 78.04, an MAE of 56.30, and an R^2^ score of 0.81 when predicting TDS. Similarly, for EC predictions, the reduced model achieved an RMSE of 81.26, an MAE of 69.17, and a R^2^ score of 0.87 (see Table 4).

**Table 4:** Performance of the DT model with reduced variables.

ML model	TDS			EC		
	RMSE	MAE	R^2^	RMSE	MAE	R^2^
DT	78.04	56.30	0.81	81.26	69.17	0.87

It is important to recognize the inherent trade-offs in dimensionality reduction; some information loss is inevitable when simplifying data representations. Assessing the proportion of explained variance by the reduced feature set helps to balance model parsimony against predictive performance.

As it has been mentioned, this dimensionality reduction by using interpretability modeling, each individual prediction is assessed to identify the role of the variable in every single prediction, and the subtraction of the less important variables is done to reduce the complexity of the data and analysis. This is done through studying variable importance and then the statistical relationships with the target that was presented in the next parts.

These reduced models are particularly actionable for operational monitoring: in settings without continuous EC/TDS sensors, the 4-variable DT model can estimate salinity from routinely gauged Discharge plus targeted ion sampling (e.g., quarterly Na/Cl), achieving ~ 85–90% of full-model skill at substantially lower cost.

### Interpretability

**Fig. 5:**
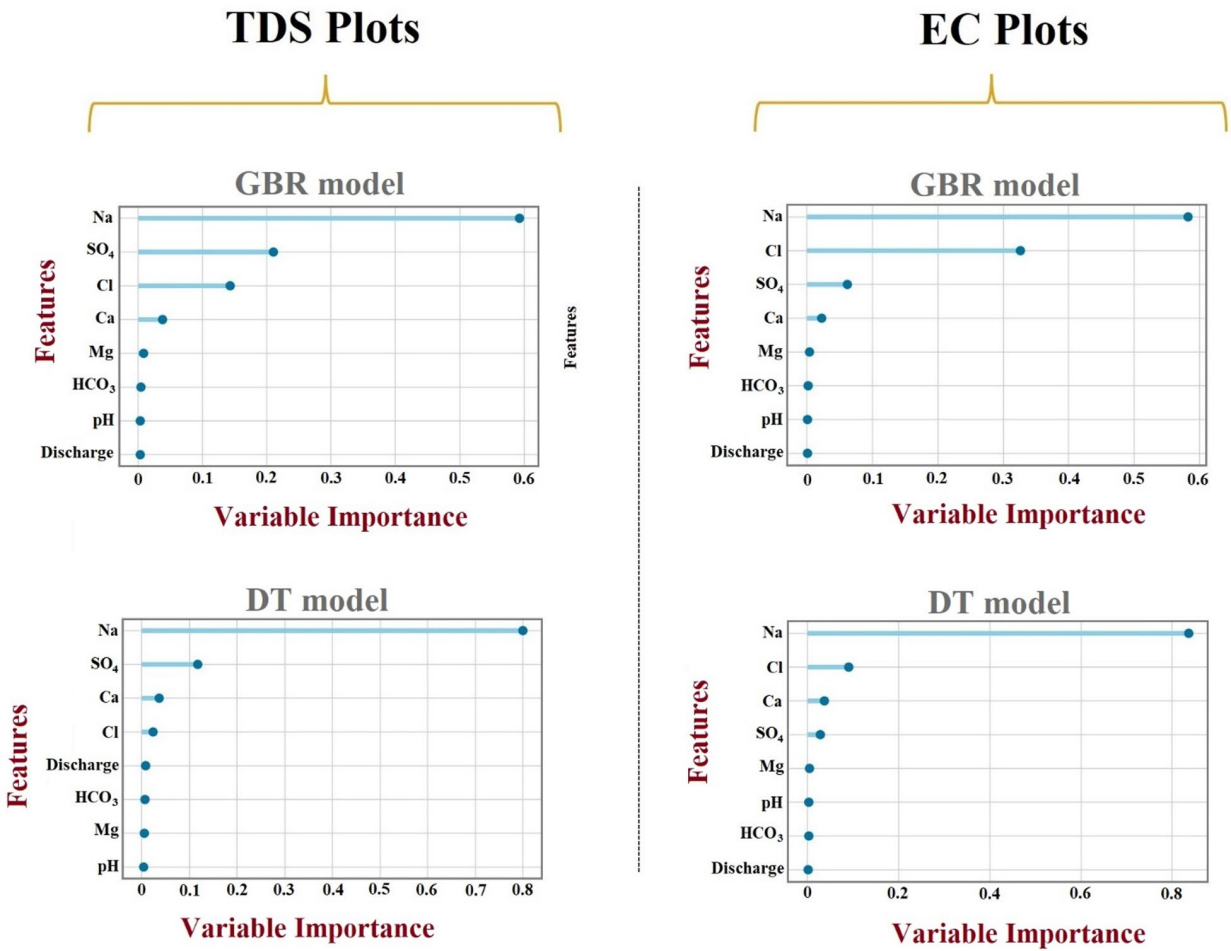
Variable importance plot of the quantitative and qualitative components on TDS and EC in ML models.

The variable importance of TDS and EC for the GBR and DT models is presented in Fig. 5. These models were selected because the GBR model was the best model in terms of accuracy, and the DT model was the best model in terms of dimensionality reduction. In that sense, in DT, we can have the highest reduction in the number of features yet accurate enough to predict the target. In these plots, an importance indicator indicates how much each variable contributes to the model prediction. Based on the results, which are depicted in the figure, for TDS in both models, Na and SO_4_ have the greatest contribution to the prediction, and Discharge and pH for the GBR model, and pH and Mg for the DT model have the least effect on the prediction. However, Na and Cl have the most influence in both models on EC prediction, and Discharge and pH for the GBR model and Discharge and HCO_3_ for the DT model have the least influence on EC.

**Fig. 6:**
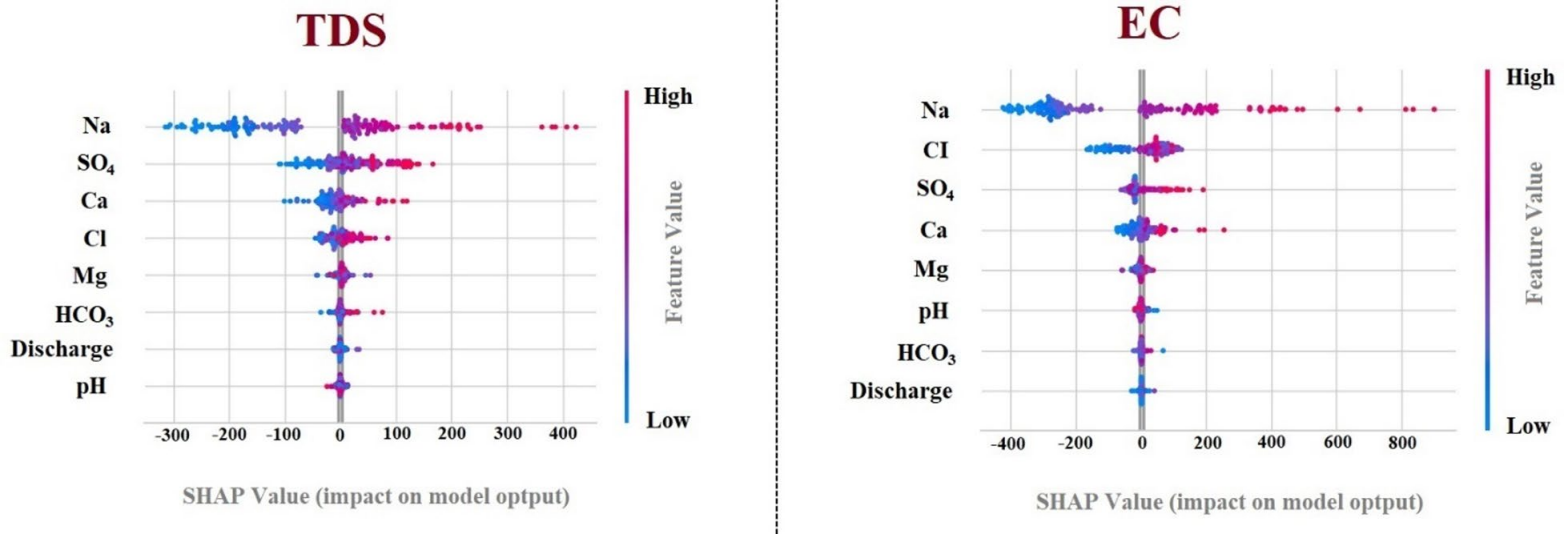
The variable importance of the quantitative and qualitative components (SHAP value) on TDS and EC according to the DT model.

The SHAP value diagram and the importance of the quantitative and qualitative contribution of each variable on TDS and EC predictions using based on the DT model are shown in Fig. 6. It should be mentioned and pointed out that this SHAP value plotting does not illustrate the “Causality” but rather it shows the “Contributions of the predictors in the predictions” which means that how each variable’s value has contributed to every single prediction. For TDS, the importance rankings show Na and SO_4_ as the most influential predictors, with pH, Discharge, HCO_3_, and Mg contributing least. For EC, Na and Cl dominate, while Discharge, HCO_3_, and pH are of minor importance. These patterns are consistent between gain-based importance and SHAP values, and they align with independent causal analysis on the same dataset, which identified Na, SO_4_, Cl, Mg, and Ca as key positive causal drivers of TDS in the previous study in the Karkheh basin^38^. The summary plot (Fig. 6) highlights that some of the variables (e.g., Discharge, pH, HCO_3_ for EC, which are close to 0) were less significant in predicting the output, while Na, SO_4_, and Cl had a significant contribution to the output (EC).

SHAP analysis confirms Na and Cl as the strongest positive associations with TDS/EC predictions, with Discharge showing a predominantly negative influence (especially at high values, consistent with dilution). These patterns align directionally with causal inference on the same dataset, where Na, Cl, SO_4_, Mg, and Ca exert significant positive causal effects on TDS (back-door adjustment, *p* < 0.05), while pH and HCO_3_ show negative effects^38^. Such associations validated against causal evidence reframe the models as tools for prioritizing hydrologically meaningful predictors in data-sparse monitoring.

### Hydrological interpretation

**Fig. 7:**
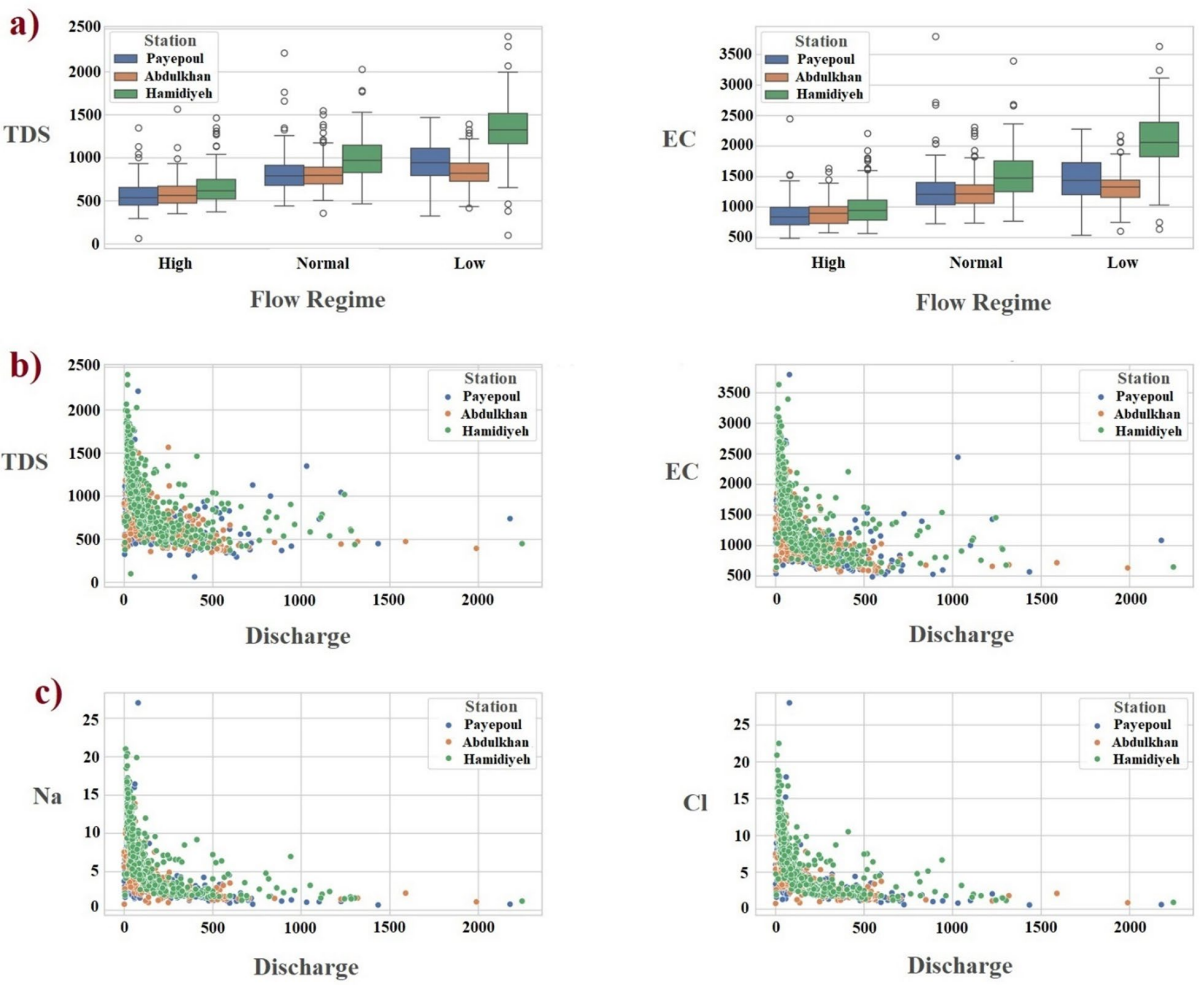
(**a**) Boxplots of TDS and EC under different flow regimes (Low, Normal, High) for three hydrometric stations along the Karkheh River, and (**b**) Scatter plots showing the relationship between river Discharge and (**c**) key water quality variables (TDS, EC, Na, Cl) at three hydrometric stations.

The dominant contributions of Na and Cl to TDS and EC prediction, as revealed through SHAP analysis, are consistent with prior causal analyses identifying Na and Cl as key positive influences on TDS, alongside Discharge’s negative dilution association^38^. In other words, for TDS, Na and SO_4_ are the main contributors in the ML models, whereas Na and Cl dominate EC, with all three ions showing higher concentrations during low-flow, baseflow-dominated periods. These ions, commonly associated with baseflow or anthropogenic input, act as reliable tracers for salinization processes. In contrast, Discharge demonstrates a mostly negative SHAP influence, especially at high flows, indicating the role of hydrological dilution. These relationships are consistent with observed trends: Na and Cl concentrations increase TDS/EC, while higher Discharge correlates with lower salinity due to dilution effects. This interpretation reframes our study not merely as an exercise in predictive modeling but as a data-driven exploration of underlying hydrological processes. In doing so, we demonstrate that interpretable ML models can be harnessed to extract mechanistic understanding from complex water quality datasets. As can be seen in the Fig. 7a (the box plots), flow regimes were classified based on the 25th and 75th Discharge percentiles. The results highlight significantly higher TDS and EC concentrations during low-flow periods, consistent with reduced dilution and increased solute accumulation under baseflow or dry-season conditions^39,40^. These hydrological patterns reinforce the ML finding that Discharge has a negative association with salinity indicators. Also, the scatter plot in the Fig. 7b, c shows that a negative trend is observed, where lower Discharge corresponds to elevated concentrations of salinity-related ions and conductivity. This behavior supports the ML-inferred importance of Na and Cl and the inverse role of Discharge in predicting TDS and EC, illustrating the hydrological control of flow conditions on solute dynamics.

Flow-regime boxplots (Fig. 7a) show elevated TDS/EC under low flows, associated with reduced dilution and higher conservative ion concentrations (Na and Cl) patterns consistent with causal drivers identified in prior analysis of this basin^38^. Scatterplots (Fig. 7b, c) reinforce the negative Discharge-salinity association across stations.

### Anomaly hydrological events

**Fig. 8:**
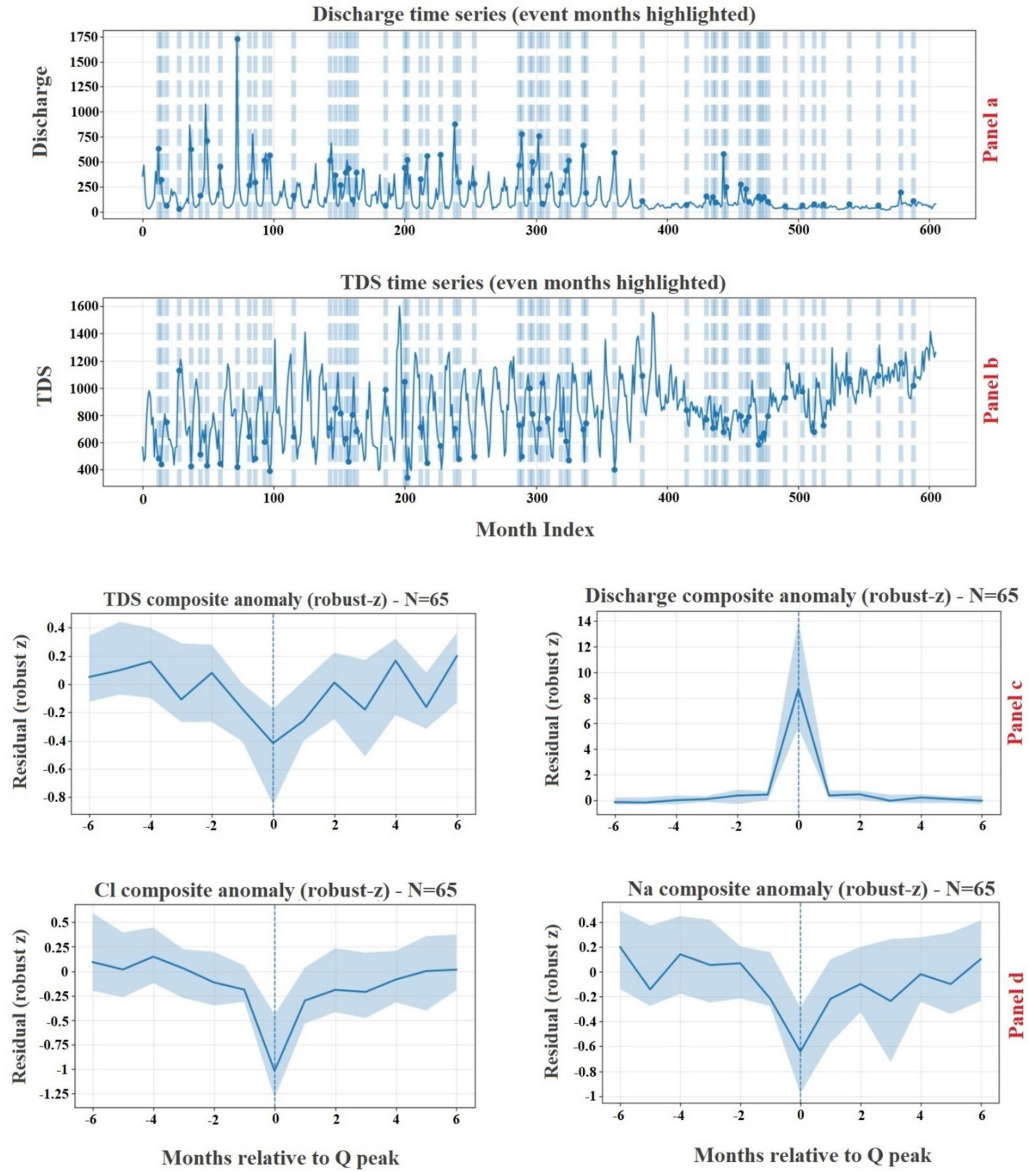
Hydrologic-event anomalies and multivariate composites in the Karkheh River (1968–2018). Discharge (**a**) and TDS (**b**) time series with detected event months (blue bands). TDS and Discharge (**c**) and Cl and Na (**d**) event-centred composites (robust z) aligned to the Discharge peak (t = 0); line = median, shading = IQR. TDS/Cl/Na dip at t = 0 (flood dilution) then recover.

This section summarizes how salinity responds to short-lived hydrologic anomalies and multivariate composites in Fig. 8. Panels a and b show the monthly Discharge and TDS series with event months (vertical bands) identified on STL-detrended residuals using a robust-z threshold (z ≥ 2). Across 1968–2018, this yields *N* = 65 Discharge spikes. Panels c and d present event-centered composites (median residuals, robust-z units) over a ± 6-month window. The composites reveal a sharp, transient Discharge peak at t = 0 followed by rapid decay, and concurrent negative excursions in TDS, Cl, and Na, indicating dilution during high-flow pulses. The median anomaly at t = 0 is largest for Cl (≈ − 1 to − 1.2 z), moderate for TDS (≈ − 0.5 to − 0.7 z), and smaller for Na (≈ − 0.2 to − 0.4 z). Bootstrap 95% envelopes remain largely below zero at the event peak, supporting a consistent dilution signature across events despite inter-event variability. Recovery is ion-specific. TDS returns toward baseline within ~ 2–3 months, Cl often rebounds faster (≈1–2 months) and shows a mild post-event overshoot, while Na exhibits broader uncertainty and a more protracted recovery, consistent with slower source/mobilization dynamics or exchange processes. The amplitude ordering (|Cl| >|TDS| >|Na|) and the short recovery of Cl point to rapid mixing and flushing during flow surges, whereas the lingering Na response suggests partial decoupling from instantaneous dilution. Together, these patterns indicate that episodic high-flow events are predominantly dilution-dominated, temporarily depressing dissolved ions before returning toward background levels, with ion-specific hysteresis and recovery times. Operationally, the composites imply that intensified sampling around t ∈ [− 1, + 2] months captures the bulk of anomaly dynamics, and that ion-specific triggers (stronger for Cl, earlier for TDS, longer tail for Na) can inform event-aware monitoring and early-warning thresholds. Operationally, this implies that EC sensors—while valuable—need not be deployed continuously; instead, the framework identifies optimal windows (e.g., t =  + 1–2 months post-peak for TDS recovery) for cost-effective grab sampling, maximizing information gain from limited budgets.

### Limitations and future works

This study presents an interpretable ML framework that integrates long-term hydrological and water quality data to elucidate the dominant drivers of salinity, specifically TDS and EC in arid river systems. While the Karkheh River offers a valuable case due to its data richness and regional importance, further validation across multiple river basins would be essential to generalize the approach.

A key strength of this study lies in its ability to maintain model interpretability while reducing data dimensionality. The integration of SHAP analysis confirmed that conservative ions (Na and Cl) and Discharge are not only statistically important but hydrologically meaningful indicators of salinity dynamics. These insights are especially relevant for semi-arid regions increasingly impacted by climate variability, groundwater extraction, and urban pressures.

However, some limitations remain. First, results come from single river systems and this is important to ensure if it is still feasible to other locations with different climatic and geological conditions. The study is process-oriented for this region and multi-catchment testing would be a logical next step. Also, while the reduced-input models preserved predictive accuracy (Figs. 5, 6), they were built on observed data and do not explicitly incorporate future climate or land use scenarios. Consequently, their predictive reliability under non-stationary conditions requires further exploration. Second, this study focused on monthly data; finer temporal resolution (e.g., daily or event-based) may reveal transient responses to storms or anthropogenic Discharges that are masked at coarser scales. While SHAP provides consistent feature associations, causal claims require independent validation, here provided via back-door adjustment. Future work will integrate structural causal models to further disentangle confounders. Another limitation is The event-based composite approach assumes that events with similar discharge anomalies have comparable hydrochemical responses, which may hide important event-to-event variability (e.g. different source areas, storm tracks, land-use patches).

Future work should aim to:

- Extend this framework to other river systems under diverse hydro-climatic regimes,
- Integrate physically based hydrological modeling to complement the data-driven findings,
- Explore causal inference or hybrid models to strengthen process attribution, and
- Link predictive outputs directly to decision-support tools for real-time monitoring or adaptive water quality management.

Ultimately, the synergy between hydrological understanding and interpretable ML offers a promising pathway for informed, robust, and resource-efficient water quality management under growing environmental pressures.

## Conclusion

This study applied an interpretable ML framework to a 50-year record of monthly Discharge, major ions, and pH from three stations on the Karkheh River in Iran to investigate long-term salinity dynamics in an arid river system. By coupling nonlinear learners with time-aware train-test splitting, the models achieved high predictive skill for TDS and EC while avoiding overfitting to the multi-decadal record. Feature attribution and variable-importance analyses consistently highlighted Na and Cl as the dominant contributors to TDS and EC, whereas river Discharge exerted a largely negative influence that reflects its dilution role during high-flow conditions. These patterns were reinforced by the flow-regime analysis, which showed systematically higher TDS, EC, and key ion concentrations under low-flow conditions and marked reductions during high-flow periods, indicating the combined effects of reduced dilution, groundwater contributions, and anthropogenic inputs during dry phases. Dimensionality-reduction experiments demonstrated that a strongly simplified model based on a subset of predictors can retain most of the predictive skill of more complex models. In particular, a four-variable decision-tree configuration preserved acceptable accuracy for both TDS and EC, suggesting that a minimal sensor package focusing on the most informative ions and Discharge can provide cost-efficient salinity monitoring in other data-limited arid rivers. This result is directly relevant for agencies facing budget constraints on continuous multi-parameter monitoring. The event-centered composite analysis of detrended residuals across multiple Discharge spikes further revealed coherent, short-lived dilutions of TDS, Na and Cl at flood peaks, followed by ion-specific recovery timescales from roughly one to several months. These findings imply that intensified sampling during, and shortly after, high-flow events can capture the bulk of salinity anomaly dynamics, and that thresholds and alerts could be designed to be regime- and event-aware rather than relying solely on fixed concentration limits. While the use of monthly grab-sample data limits the ability to resolve sub-daily salinity excursions, the results show that interpretable ML combined with flow-regime and event analyses can extract mechanistic insight and insights for operational monitoringfrom the long-term archives that dominate many water-quality networks. Extending this framework to other basins, exploring higher-frequency datasets, and coupling with process-based models or scenario analyses would further strengthen its role in supporting adaptive, cost-effective salinity management under growing climatic and anthropogenic pressures.

## Supplementary Information

Below is the link to the electronic supplementary material.

Supplementary Material 1

## Data Availability

The authors appreciate the Ministry of Energy, Iran, for supplying the required data for the real-case studies described in this paper. The data has been given by local authorities, thus the data will be available upon request.
