# Supplementary material for: Interpretable machine learning for river salinity dynamics in arid basins
Source: Sci Rep. 2026 Apr 20;16:18310. doi: 10.1038/s41598-026-49042-9 (PMC13260926; doi:10.1038/s41598-026-49042-9)
Supplement: Supplementary file 1 — Supplementary Material 1 [file 41598_2026_49042_MOESM1_ESM.docx]

**S. 1. Multi-Layer Perceptron (MLP) model**

MLP models were employed to predict water quality indices, using the features including pH, Mg, Na, Cl, and others according to the predictive indicators selected by correlation matrix to predict the target which is “TDS” and “EC”. The training method (optimizing algorithm) for this method is “Limited memory Broyden Fletcher Goldfrab Shanno” which is shown as “lbfgs”. “lbfgs” is a quasi-Newton method that approximates the Hessian matrix using past gradient information. Each architecture includes an input layer, one or many hidden layers, and an output layer. In order to assess the performance of each architecture, the least Mean Squared Error (MSE) and R^2^ were chosen. The Grid Search algorithm has been employed to identify the optimum architecture to avoid overfitting which is common problem for this training method. The optimum architecture of the MLP is presented in table 1. Every single hyper-parameter is playing a role in order to achieve local optima. As an example, the activation function that is used to translate the input layer multiplied by weight factor to output layer. As an example, the equation for “tanh” is $\tanh x=\frac{(e^{x}-e^{(-x)})}{(e^{x}+ e^{(-x)})}$, which $x$ is the input value to the function, $e^{x}$ represents the exponential function of $x$ and $e^{(-x)}$ is the exponential function of $-x$, and it approaches zero when the input increases. Alpha is used for regularization and to combat the overfitting problem. The greater number of nodes and layers necessarily does not mean there is an increase in the accuracy, vice versa, it is possible to cause the overfitting. Thus, it is important to have an optimum value [1, 2].

| **Activation function** | **Alpha** | **Hidden layer size** | **Solver** |
| --- | --- | --- | --- |
| tanh | 0.001 | 10 | lbfgs |

**Table 1.** The architecture of MLP model

An example of a conventional MLP architecture is seen in Fig S.1. According to this figure, an initial prediction is set through multiplication of weight factor and input value then it is compared with the output value and in case of weak accuracy, this process is repeated until having enough accuracy.


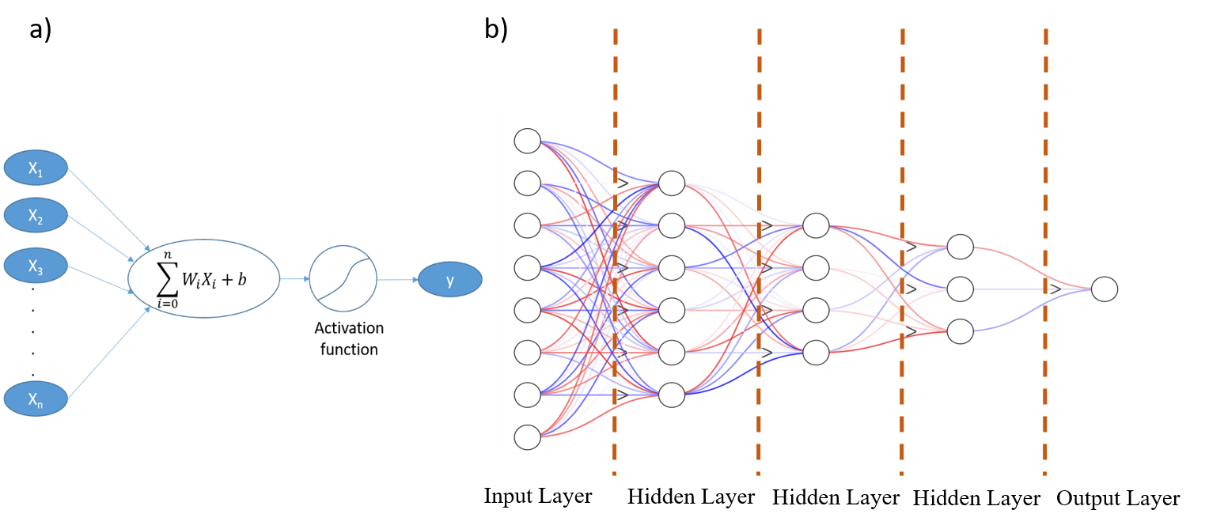


**Fig S.1.** a) The general overview of a neuron that transfers knowledge from the input layer to the output layer in MLP model. b) An example of an architecture of MLP that in this figure the number of hidden layers is 3 however this is just a general scheme.

**S. 2. Random_Forest (RF) and Decision_Tree (DT) models**

In general, ensemble learning models are the models that combines several base models in order to produce one optimal predictive model. RF combines the output of multiple DT to reach a single result. In other words, the RF is made up of multiple decision trees with basic questions that the answers to the questions will lead you to a better decision or result [3]. In table 2, the design configuration for RF and DT models is presented. In both, the quality of making decisions to split and make the rest of the forest or tree, the function that considers this quality control is “Criterion”. “Max depth” is an integer number that shows the number of times that the tree or forest grows. The strategy to split the tree in this case is “Best”. Finally, the “ccp-alpha”, the higher value of cp leads to a smaller tree. A too small value of cp leads to overfitting and a too large cp value will result to a too small tree. Also, Fig S.2 below shows an example of how to do the work in models. The criterion has been chosen is “squared_error” which is used to measure the impurity of a node and guide the decision tree’s splitting process. It essentially combines two concepts: a) Minimizing the MSE, and b) Focusing on the improvement of the performance of the model in the prediction. This goes beyond simply calculating MSE while in every step it tries to reduce MSE that is achieved in previous steps.

| **Criterion** | **Max depth** | **Min_sample_leaf** | **Min_sample_split** | **ccp_alpha** |
| --- | --- | --- | --- | --- |
| Squared error | 5 | 5 | 10 | 0.001 |

Table 2. The architecture of RF and DT models


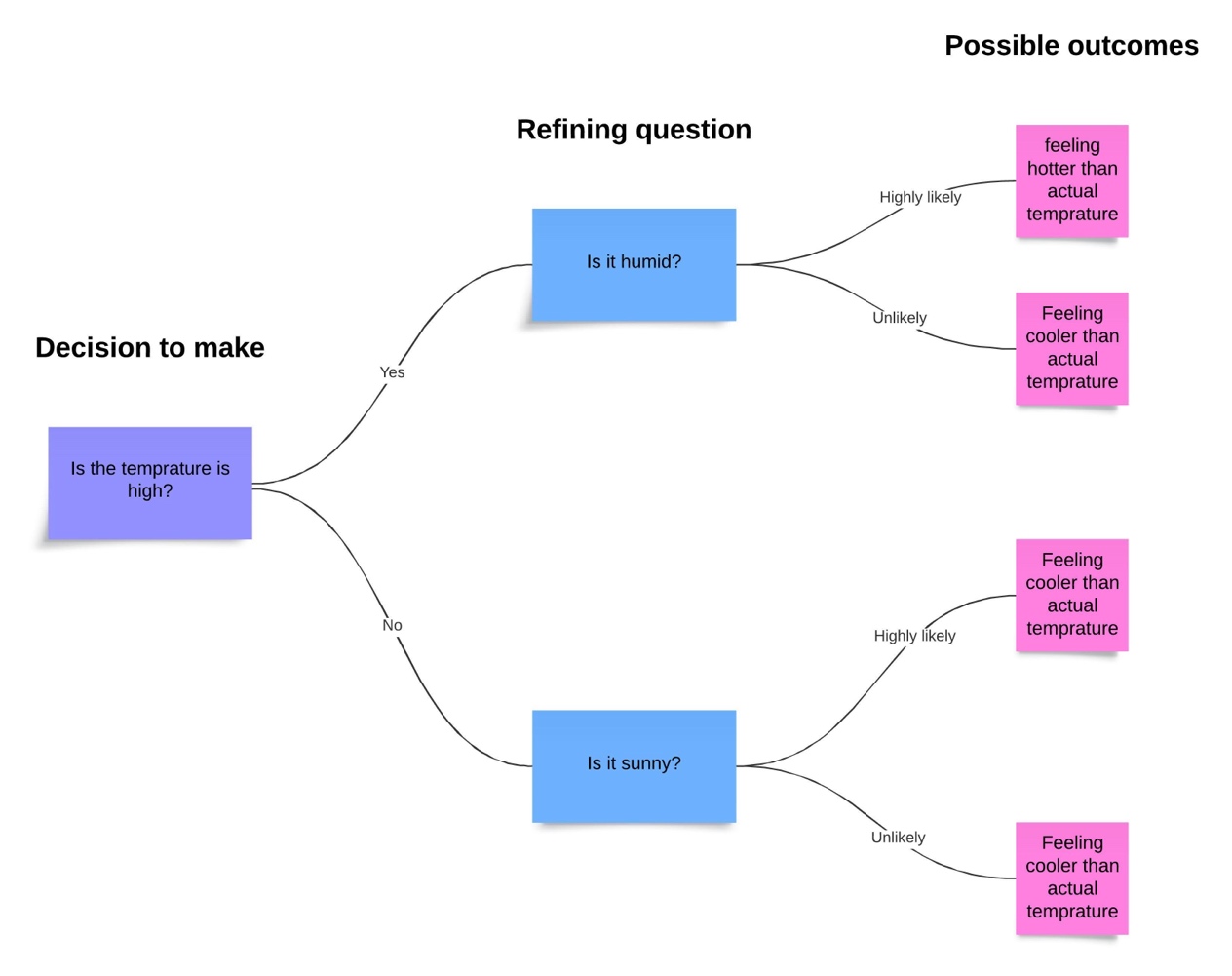


**Fig S.2.** An example schematic view of the process of processing RF and DT models

**S. 3. K_Nearest_Neighbors (KNN) model**

The KNN is a non-parametric, supervised algorithm that considers all data to compare the proximity. The basic is simply trying to explore the neighbourhood. In this algorithm, the machine presumes that similar values appear in their vicinity. The versatility of the method caused scientists to implement numerous problems using this method. Also, from the fact that this model, is being tunned easier than other methods because of having less factors, it would become more popular [4]. Amongst the variables, some of them have been given in the table 3. The first, as it is expected, is the “number of neighbors” which is the number of cases in the neighborhood of the values that are considered. In weighted KNN, the nearest K points are given a weight using a function called the kernel function. The value of “P” in Minkowski distance is tunned based on experimentation and trial and error. The usual value of P is either 1 or 2 for most problems. The exact value depends on the applications [5].

Table 3. The architecture of KNN model

| **Number of neighbours** | **Weight** | **Algorithm** | **P** | **Metric** |
| --- | --- | --- | --- | --- |
| 7 | distance | Auto | 2 | Minkowsiki |

**S. 4. eXtreme Gradient Boosting (XGBoost) model**

XGBoost, denoting “eXtreme Gradient Boosting”, stands out due to its optimized and parallelizable nature compared to the other decision-based models. XGBoost gives an efficient implementation of the gradient boosting algorithm. XGBoost can be applied directly for predictive regression modelling [6, 7]. Minimizing the loss function throughout the training and learning phase is the aim of XGBoost. It gives details on how close the actual and expected numbers are to one another.

“Min child weight” defines minimum sum of weights of all observations required in a child. It is the step size shrinkage used in update to avoid overfitting and usually it has a range between 0 and 1 which in this study it is 0.1. Pedregosa et al (2011) [8] and Bentéjac et al (2021) [9] introduced “Col sample”, which is a family of parameters for sub sampling of the columns. In the sense that, it randomly samples the columns (named as variables) from the data before it calculates the distance to the neighbours. In other words, it controls the percentage of variables used in each distance calculation. The architecture of XGBoost model is given in Table 4 in below.

**Table 4.** The architecture of XGBoost model

| **N. Estimator** | **Learning rate** | **Max depth** | **Objective** | **Sub sample** | **Col sample** | **Min child weight** |
| --- | --- | --- | --- | --- | --- | --- |
| 600 | 0.03 | 5 | Squared error | 0.8 | 0.8 | 5 |

**S. 5. Gradient Boosting Regression (GBR) model**

GBR is a traditional Gradient Boosting framework where the model grows sequentially, with each stage comprising a regression tree fitted on the negative gradient. It has been observed to provide competitive predictions with fewer trees compared to other boosting algorithms. However, one of its limitations includes its relatively slower performance due to the sequential nature of tree-building. There is also a potential for overfitting if tree numbers are not optimized [10]. The architecture of GBR model is shown in Table 5.

Table 5. The architecture of GBR model

| **Min sample split** | **Min sample leaf** | **Max depth** | **Learning rate** | **Number of estimators** | **Sub sample** |
| --- | --- | --- | --- | --- | --- |
| 10 | 5 | 3 | 0.05 | 300 | 0.8 |

“Learning rate”, is the shrinkage that the model does at every step. In other words, by learning rate, the contribution of each tree, shrinks. Next, is the “number of estimators” that refers to the maximum number of boosting trees (or rounds) to be used in building the model. Not all the time, but usually the higher number of trees will result in better learning. Intuitively, the more trees that are added to the model, the more complex it is. The “Max variable” is the number of variables to consider when looking for the best split. “Min sample leaf” and “Min sample split” are the minimum number of samples and sub sample required to be at a leaf node.

**S. 6. Extended data figures**

Fig S.3 shows the scatter plot of investigated components with respect to TDS and EC. As can be seen, the distribution is almost linear when Cl, SO_4_, or Mg was used as an independent variable and TDS as a dependent variable. But other variables such as Discharge, pH, HCO_3_, Ca and Na have scattered distribution which means there is no linear relationship between these and the dependent variables (TDS and EC). This shows the weakness of using linear models which in turn leads to the use of non-linear models that have the capability of dealing with this sort of data with acceptable accuracy. Therefore, non-linear models in ML were employed to assess the quality condition.


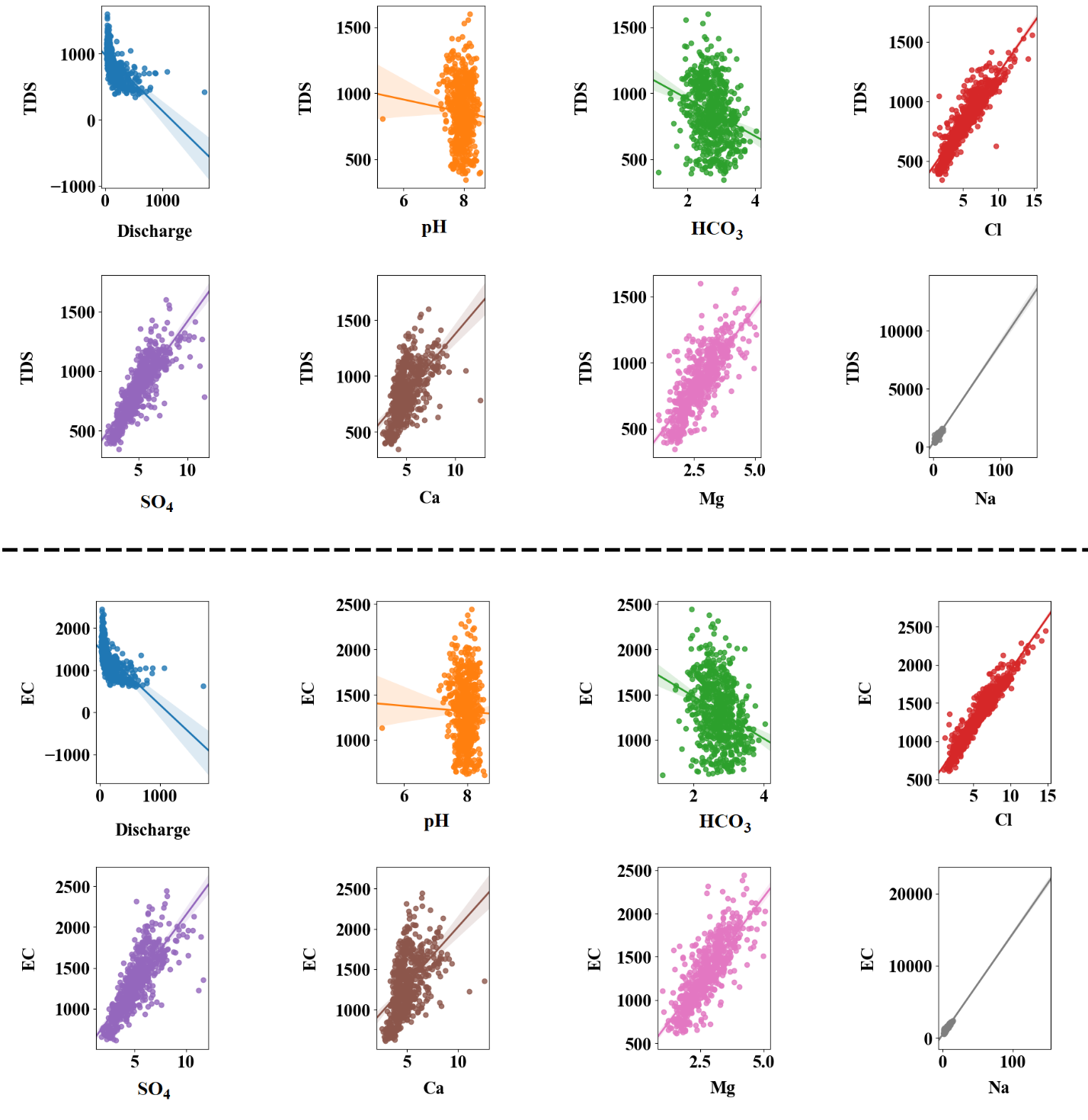
 **Fig S.3.** Scatter plot map of the quantitative and qualitative components on TDS and EC according to linear regression. The straight line in every subplot is the best fit line (linear regression line) with confidence intervals. Some of variables can not be considered using linear regression which urges to use non-linear models

Fig S.4 shows the R² distributions from rolling-origin cross-validation for the DT and GBR models predicting TDS and EC, demonstrating stable, leakage-safe performance across temporal folds compared with the main text results. Fig S.5 shows the blocked-holdout R² for simple baselines (mean, persistence, and single-variable linear models) for TDS and EC, confirming that the proposed ML models exhibit substantially higher practical forecasting skill than naive alternatives.


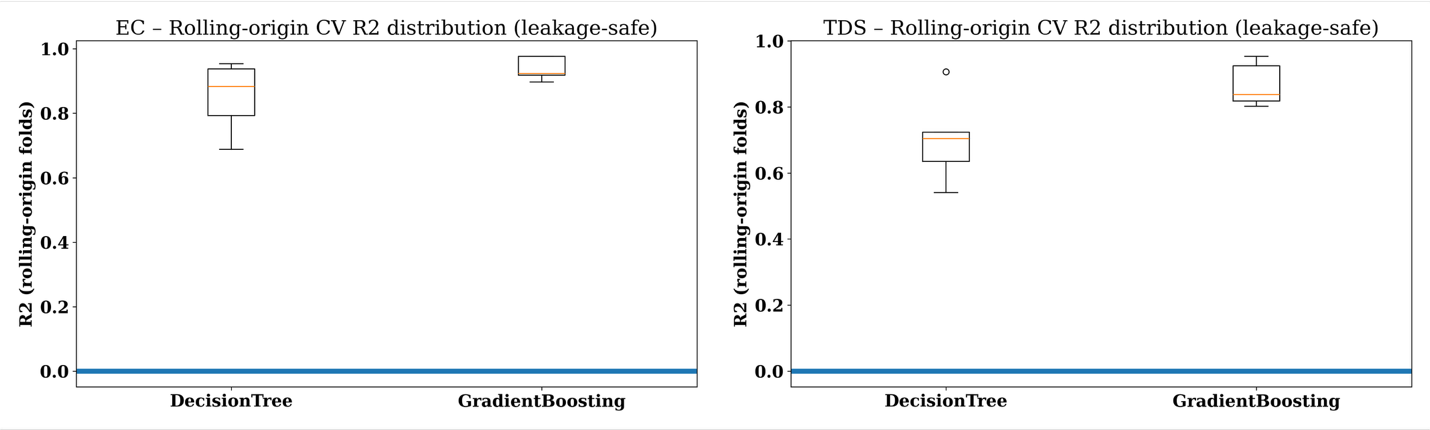


**Fig S.4.** Rolling-origin R² distribution under leakage-safe validation. R² values across sequential forward-chaining folds, where each model was trained only on past observations and evaluated on future unseen data. Consistent performance across folds confirms temporal generalization ability.


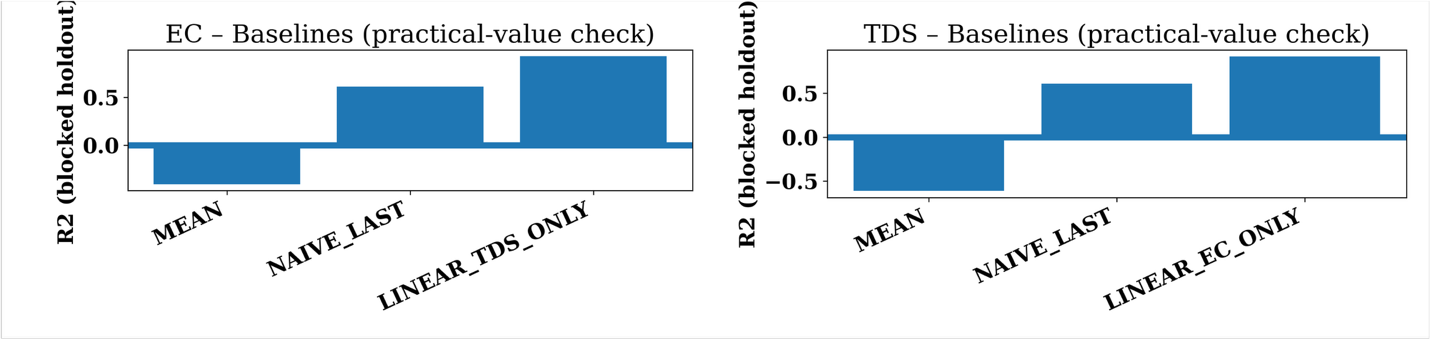


**Fig S.5.** Comparison of ML model performance against mean and persistence baselines under blocked chronological holdout evaluation.


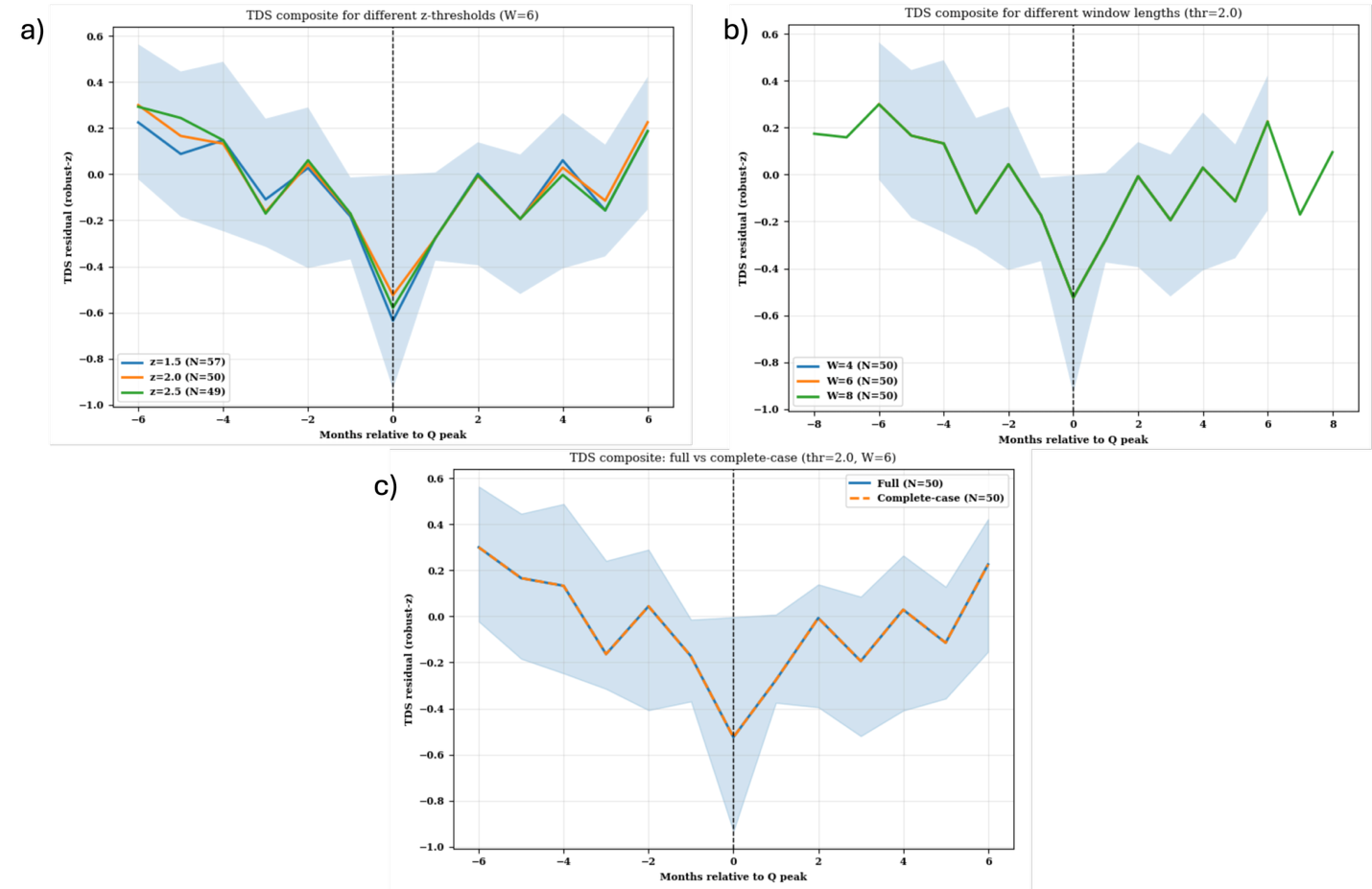


**Fig S.6.** TDS composite: Sensitivity of the discharge‑event analysis. Composites are based on monthly robust‑z residuals of TDS aligned to local maxima in discharge anomalies. (a) Effect of varying the discharge anomaly threshold, showing nearly identical TDS trajectories for events defined with z = 1.5, 2.0, and 2.5 while the number of events remains similar. (b) Effect of varying the compositing window length (±4, ±6, ±8 months), indicating that the central pre‑ and post‑peak evolution of TDS is insensitive to the chosen window, with differences confined to the outermost months. (c) Comparison of composites derived from the full dataset (including imputed months) and from complete‑case months only, demonstrating that both approaches yield virtually the same TDS response, confirming robustness to missing data and methodological choices.

**S. 7. References**

[1] Taher Shamsi, A., Menhaj, M. B. & Ahmadian, R. Estimating sediment using MLP and RBF neural networks. *Amirkabir J. Sci. Technol.* **16,** 103–110 (2006).

[2] Lam, M. Y. & Ahmadian, R. Predicting fecal-indicator organisms in coastal waters using a complex nonlinear artificial intelligence model. *J. Environ. Eng.* **149,** 04022093; 10.1061/JOEEDU.EEENG-6986 (2023).

[3] Misra, S., Li, H. & He, J. Noninvasive fracture characterization based on the classification of sonic wave travel times. In *Machine Learning for Subsurface Characterization* **4,** 243–287; 10.1016/B978-0-12-817736-5.00009-0 (2020).

[4] Hart, P. E., Stork, D. G. & Duda, R. O. *Pattern Classification* (Wiley, Hoboken, 2001).

[5] Sun, S. & Huang, R. An adaptive k-nearest neighbor algorithm. In *Proc. 7th Int. Conf. Fuzzy Systems and Knowledge Discovery* **1,** 91–94; 10.1109/FSKD.2010.5569740 (2010).

[6] Osman, A. I. A., Ahmed, A. N., Chow, M. F., Huang, Y. F. & El-Shafie, A. Extreme gradient boosting (XGBoost) model to predict the groundwater levels in Selangor, Malaysia. *Ain Shams Eng. J.* **12,** 1545–1556; 10.1016/j.asej.2020.11.011 (2021).

[7] Bojer, A. K. et al. Machine learning and remote sensing based time series analysis for drought risk prediction in Borena Zone, Southwest Ethiopia. *J. Arid Environ.* **222,** 105160; 10.1016/j.jaridenv.2024.105160 (2024).

[8] Pedregosa, F. et al. Scikit-learn: machine learning in Python. *J. Mach. Learn. Res.* **12,** 2825–2830 (2011).

[9] Bentéjac, C., Csörgő, A. & Martínez-Muñoz, G. A comparative analysis of gradient boosting algorithms. *Artif. Intell. Rev.* **54,** 1937–1967; 10.1007/s10462-020-09896-5 (2021).

[10] Otchere, D. A., Ganat, T. O. A., Ojero, J. O., Tackie-Otoo, B. N. & Taki, M. Y. Application of gradient boosting regression model for the evaluation of feature selection techniques in improving reservoir characterisation predictions. *J. Pet. Sci. Eng.* **208,** 109244; 10.1016/j.petrol.2021.109244 (2022).
